# Poor Synchronization to Musical Beat Generalizes to Speech†

**DOI:** 10.3390/brainsci9070157

**Published:** 2019-07-04

**Authors:** Marie-Élaine Lagrois, Caroline Palmer, Isabelle Peretz

**Affiliations:** 1 International Laboratory for Brain, Music, and Sound Research, Montreal, QC H3C 3J7, Canada; 2 Department of Psychology, University of Montreal, Montreal, QC H3C 3J7, Canada; 3 Department of Psychology, McGill University, Montreal, QC H3A 1B1, Canada

**Keywords:** beat deafness, music, speech, entrainment, sensorimotor synchronization, beat-finding impairment, brain oscillations

## Abstract

The rhythmic nature of speech may recruit entrainment mechanisms in a manner similar to music. In the current study, we tested the hypothesis that individuals who display a severe deficit in synchronizing their taps to a musical beat (called beat-deaf here) would also experience difficulties entraining to speech. The beat-deaf participants and their matched controls were required to align taps with the perceived regularity in the rhythm of naturally spoken, regularly spoken, and sung sentences. The results showed that beat-deaf individuals synchronized their taps less accurately than the control group across conditions. In addition, participants from both groups exhibited more inter-tap variability to natural speech than to regularly spoken and sung sentences. The findings support the idea that acoustic periodicity is a major factor in domain-general entrainment to both music and speech. Therefore, a beat-finding deficit may affect periodic auditory rhythms in general, not just those for music.

## 1. Introduction

Music is quite unique in the way it compels us to engage in rhythmic behaviors. Most people will spontaneously nod their heads, tap their feet, or clap their hands when listening to music. In early infancy, children already show spontaneous movements to music [1]. This coupling between movements and music is achieved through entrainment. Entrainment can be broadly defined as the tendency of behavioral and brain responses to synchronize with external rhythmic signals [2,3]. Currently, the predominant models of entrainment are based on the dynamic attending theory (DAT) [2,4,5,6]. According to this theory, alignment between internal neural oscillators and external rhythms enables listeners to anticipate recurring acoustic events in the signal, allowing for maximum attentional energy to occur at the onset of these events, thus facilitating a response to these events [2]. Multiple internal oscillators that are hierarchically organized in terms of their natural frequency or period are likely involved in this process. Interaction of these oscillators would permit the extraction of regularities in complex rhythms that are periodic or quasi-periodic in nature, such as music [7,8,9]. Of note, entrainment to rhythms, as modeled by oscillators, would apply not only to music but also to speech [10,11,12,13,14,15,16,17,18].

The periodicities contained in musical rhythms typically induce the perception of a beat, that is, the sensation of a regular pulsation, on which timed behaviors are built [19]. Simple movements in response to beat perception, like taps, are usually produced within a few tens of milliseconds of the beat onset, indicating the precision of the temporal predictions made about the timing of upcoming beats [20,21,22]. Listeners can extract the beat from various complex rhythms, without the need for a one-to-one correspondence between acoustic events and beat occurrences [23,24,25,26,27] and across a large range of tempi (~94–174 beats per minute) [20,28,29,30,31]. Beat extraction is also robust to moderate tempo fluctuations [8,32,33]. Beat induction from music has in fact been proposed as one of the fundamental and universal traits of music [34,35].

Musical meter, which corresponds to the hierarchical organization of beats, where some beats are perceived as stronger than others, leads to higher-order periodicities of strong and weak beats (for example, a march versus a waltz). Similarly, speech has a hierarchically organized temporal structure, with phonemes, syllables, and prosodic cues, each occurring at different time scales [16,36,37,38]. As in music, metrical hierarchy in speech may rely on the occurrence of stressed or accented acoustic events, typically associated with syllables [11,17,39,40,41]. Stress patterns in speech vary and depend on different acoustic cues according to language. The meter of “stress-timed” languages, such as English, is usually clearer than the meter of “syllable-timed” languages like French [14,42]. However, regardless of the language studied, temporal intervals between stressed syllables are not as regular in speech as in music [41,43,44,45,46].

Despite this variability in the regularity of stress or beat in spoken language, individuals seem to be able to entrain to speech. Initial evidence in this regard is the finding that the timing of speech can be synchronized with a metronome [11]. Speakers can not only adapt their speech rate to match another speaker [47,48], but they also entrain to each other’s syllables rate in conversational turn taking [18,49]. In a prior study using a similar experimental design to the present study [14], French and English monolingual speakers and French–English bilingual speakers were invited to tap their finger along with the beat they perceived in French and English sentences spoken with natural prosody. The variability of intervocalic intervals (IVIs) in these sentences predicted the participants’ inter-tap variability, suggesting that the participants were able to entrain to the speech stimuli.

While there is evidence of entrainment to speech, a puzzling difference exists between the absence of synchronous (“choral”) speech and the widespread and exquisite synchronization observed in music. To address this issue, Cummins [50,51] proposed that synchronous speech should be possible because (1) speakers of the same language have mastered the association between motor actions and speech sounds of their language, and (2) they share knowledge of speech timing. He supports his claim by showing that speakers can synchronize while reading an unfamiliar text without prior practice, which the author considered an indication of aperiodic synchronization [10,52,53,54]. According to this perspective, entrainment to speech and music would reflect a fundamental propensity of humans to time their actions with the rhythm of an external event.

Entrainment to speech and music has rarely been compared behaviorally, with few previous studies in this regard. In one of these [55], the influence of music and speech on entrainment was assessed through interference. The main task was to synchronize finger taps to a metronome while hearing highly isochronous computer-generated music or regularly spoken poems. When the metronome tones and the musical beats or stressed syllables were perfectly aligned, higher variability in the asynchronies between taps and metronome was found with the speech distractor compared to the musical one. When misaligned, both music and speech led to synchronization interference by increasing the asynchrony between taps and metronome onsets, and music induced the largest asynchrony. In a second experiment in this study, the stimuli were better matched: songs, either sung with lyrics, sung with a single syllable, or spoken with a regular pace, were presented. In this case, misaligned stimuli had identical detrimental effects on the variability of tapping to the metronome, whether spoken or sung. Therefore, when isochrony is equalized between music and speech, entrainment appears to be very similar.

However, natural speech is typically not isochronous. In a second study comparing music and speech [56], using the same paradigm as the current study, native French and English speakers tapped along with French and English sentences in three conditions: naturally spoken, regularly spoken, and sung with a simple melody. The inter-tap intervals (ITIs) were more variable in the naturally spoken sentences than in the other conditions. The taps were also more closely aligned to the beat (the nearest implied metronome click to which the singer synchronized her renditions of the stimuli) for sung than for regularly spoken sentences. These results show an overall effect of regularity on entrainment, with music being more suitable to elicit entrainment than regular speech.

Here, we tested the same materials as those used by Lidji and collaborators [56] with individuals who have a documented deficit in tracking the beat in music. This disorder is characterized by an inability to synchronize whole-body movements, clapping, or tapping to the beat of music [57,58,59,60,61], to amplitude-modulated noise derived from music [60], and to metronome-like rhythms [62,63]. This beat-finding deficit occurs in the absence of intellectual disability or acquired brain damage. Study of this “beat-deaf” population provides an opportunity to test the domain specificity of entrainment mechanisms. If the beat-finding disorder initially diagnosed with music also disrupts entrainment to speech, then the association will provide evidence for the domain-general nature of entrainment mechanisms to auditory rhythms.

Beat-deaf individuals and matched control participants who did not exhibit a beat processing disorder were asked to tap to spoken and sung sentences. If entrainment abilities are domain-general, then beat-deaf participants should show deficits to adapt their tapping period to the intervocalic period between syllables to all versions of sentences, compared to the control group. The control group was expected to replicate the findings of [56] showing largest inter-tap interval variability to natural speech, next largest to regularly spoken sentences, and smallest inter-tap variability to sung sentences, and with more accurate synchronization to the intervocalic period between syllables of sung sentences than regularly spoken sentences. Alternatively, if entrainment is domain-specific, beat-deaf participants’ tapping should be most impaired for sung sentences and unimpaired (meaning similar to the control group) for speech.

## 2. Materials and Methods

### 2.1. Participants

Thirteen beat-deaf French-speaking adults (10 females) and 13 French-speaking matched control participants (11 females) took part in the study. The groups were matched for age, education, and years of music and dance training (detailed in Table 1). One beat-deaf participant was completing an undergraduate degree in contemporary dance at the time of testing. Accordingly, a trained contemporary dancer was also included in the control group. All participants were non-musicians and had no history of neurological, cognitive, hearing, or motor disorders. In addition, all had normal verbal auditory working memory and non-verbal reasoning abilities, as assessed by the Digit Span and Matrix Reasoning subtests of the WAIS-III (Wechsler Adult Intelligence Scale) [64], with no differences between groups on these measures (*p*-values > 0.34; Table 1). Participants provided written consent to take part in the study and received monetary compensation for their participation. All procedures were approved by the Research Ethics Council for the Faculty of Arts and Sciences at the University of Montreal (CERAS-2014-15-102-D).

#### Procedure Prior to Inclusion of Participants in the Study

Participants in the beat-deaf group had taken part in previous studies in our lab [63,65] and were identified as being unable to synchronize simple movements to the beat of music. Control participants had either taken part in previous studies in the lab or were recruited via online advertisements directed toward Montreal’s general population or through on-campus advertisements at the University of Montreal.

**Figure 1:**
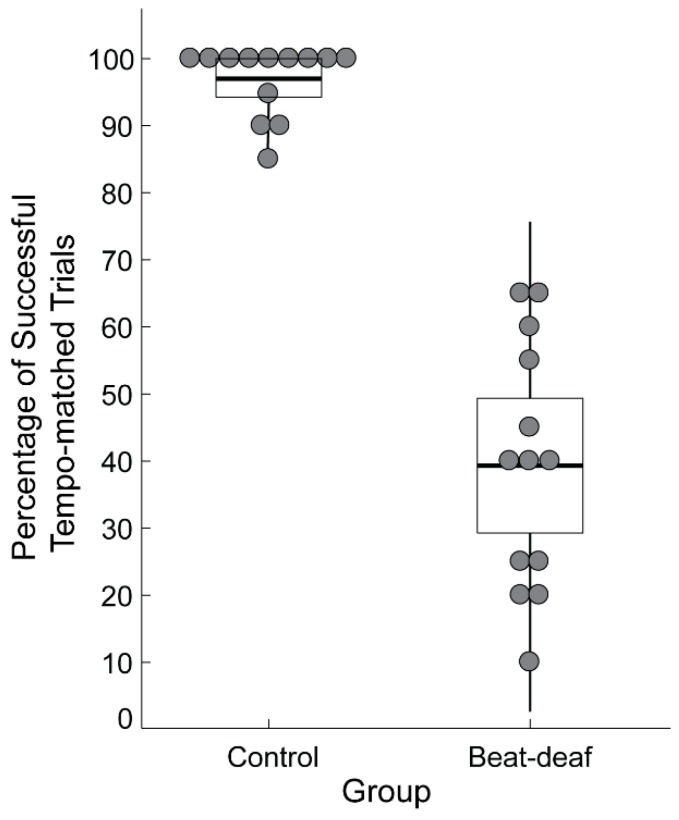
Performance of participants in the control and beat-deaf groups in the beat production task of the Montreal Beat Alignment Test (M-BAT). Each dot represents a participant. Boxes correspond to a 95% confidence interval from the mean based on the standard error of the mean (SEM). The black horizontal line within each box indicates the group mean. The vertical lines represent two standard deviations from the mean.

Inclusion in the current study was based on performance on the Montreal Beat Alignment Test (M-BAT) [66]. In a beat production task, participants were asked to align taps to the beat of 10 song excerpts from various musical genres. Tempo varied across the excerpts from 82 beats per minute (bpm) to 170 bpm. Each song was presented twice, for a total of 20 trials. Control participants successfully matched the period of their taps to the songs’ beat in at least 85% of the trials (M = 96.9%, SD = 5.2%); successful period matching was determined through evaluation of *p*-values on the Rayleigh *z* test of periodicity, with values smaller than 0.05 considered successful. In the beat-deaf group, the average percentage of trials with successful tempo matching was 39.2% (range of mean values: 10–65%, SD = 18.3%). As shown in Figure 1, there was no overlap between the groups’ performance on this task, confirming that the participants in the beat-deaf group showed a deficit in synchronizing their taps to the beat of music.

Prior to their participation in the current study, participants completed the online test of amusia to screen for the presence of a musical pitch perception impairment [67]. The online test is composed of three tests: Scale, Off-beat, and Off-key. The Scale test requires the comparison of 30 pairs of melodies that differ by an out-of-key note in half of the trials. The Off-beat and Off-key tests consist of the detection of either an out-of-time or an out-of-key note, respectively. A score lying 2-SD below the mean of a large population on both the Scale and Off-key tests indicates the likely presence of pitch deafness (also called congenital amusia) [67,68]. Based on the data from Peretz and Vuvan [67], a cut-off score of 22 out of 30 was used for the Scale test and 16 out of 24 for the Off-key test. Table 2 indicates the individual scores of beat-deaf participants on the online test. Half of the beat-deaf group scored at or below the cut-off on both the Scale and Off-key tests. As these cases of beat-deaf participants could also be considered pitch-deaf, the influence of musical pitch perception will be taken into account in the analysis and interpretation of the results. All control participants had scores above the 2-SD cut-offs.

### 2.2. Stimulus Materials

**Figure 2:**
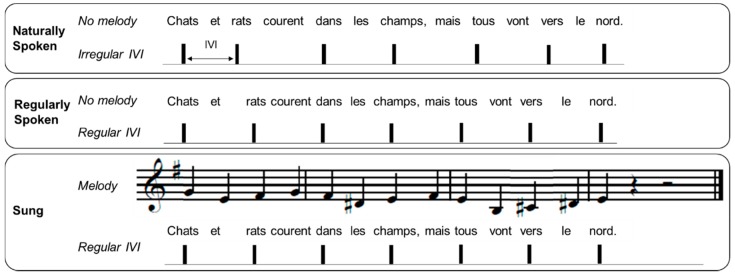
Example of a sentence in the naturally spoken, regularly spoken, and sung conditions. IVI refers to the intervocalic interval between stressed syllables.

The 12 French sentences used in this experiment were taken from Lidji et al. [56]. Each sentence contained 13 monosyllabic words and was recorded in three conditions as depicted in Figure 2. The recordings were made by a native Québec French/English female speaker in her twenties who had singing training. Recordings were made with a Neumann TLM 103 microphone in a sound-attenuated studio. In the naturally spoken condition, the speaker was asked to speak with a natural prosody (generating a non-periodic pattern of stressed syllables). In the regularly spoken condition, sentences were recorded by the speaker to align every other syllable with the beat of a metronome set to 120 bpm, heard over headphones. In the sung condition, the sentences were sung by the same speaker, again with every other syllable aligned to a metronome at 120 bpm, heard over headphones. Each sung sentence was set to a simple melody, with each syllable aligned with one note of the melody. Twelve unique melodies composed in the Western tonal style in binary meter, in major or minor modes, were taken from Lidji et al. [56]. These melodies were novel to all participants. Although each sentence was paired with two different melodies, participants only heard one melody version of each sung sentence, counterbalanced across participants.

Additional trials for all three conditions (naturally spoken, regularly spoken, sung) were then created from the same utterances at a slower rate (80% of original stimulus rate, i.e., around a tempo of 96 bpm) using the digital audio production software Reaper (v4.611, 2014; time stretch mode 2.28 SOLOIST: speech, Cockos Inc., New York, United States). This ensured that the beat-deaf participants adapted their taps to the rate of each stimulus and could comply with the task requirements. All the stimuli were edited to have a 400 ms silent period before the beginning of the sentence and a 1000 ms silent period at the end of the sentence. Stimuli amplitudes were also equalized in root mean square (RMS) intensity. Preliminary analyses indicated that all participants from both groups adapted the rate of their taps from the original stimulus rate to the slower stimulus rate, with a Group × Material (naturally spoken, regularly spoken, sung) × Tempo (original, slow) ANOVA on mean inter-tap interval (ITI) showing a main effect of Tempo, *F*(1,24) = 383.6, *p* < 0.001, *ƞ^2^* = 0.94, with no significant Group × Tempo interaction, *F*(1,24) = 0.0004, *p* = 0.98 or Group × Material × Tempo interaction, *F*(2,48) = 1.91, *p* = 0.17 (mean ITI results are detailed in Table 3). Therefore, the data obtained for the slower stimuli are not reported here for simplicity.

Table 4 describes the features of the rhythmic structure of the stimuli in each condition. Phoneme boundaries were marked by hand using Praat [69], and were classified as vowels or consonants based on criteria defined by Ramus, Nespor, and Mehler [70]. Note that the analyses reported below include the stimuli at the original tempo only. Once the segmentation was completed, a MATLAB script was used to export the onset, offset, and duration of vocalic (a vowel or a cluster of vowels) and consonantal (a consonant or a cluster of consonants) intervals. The Normalized Pairwise Variability Index for Vocalic Intervals (V-nPVI), an indication of duration variability between successive vowels [71], was used to measure the rhythmic characteristics of the stimuli. A higher V-nPVI indicates greater differences in duration between consecutive vocalic intervals. Comparison of sentences in the naturally spoken, regularly spoken, and sung conditions showed a significant difference between conditions, *F*(2,22) = 21.6, *p* < 0.001, *ƞ*^2^ = 0.66. The V-nPVI was higher in the naturally and regularly spoken conditions than in the sung condition (Table 4). The coefficient of variation (CV, calculated as SD/mean) of IVIs (vowel onset to onset) is another indication of rhythmic variability [14]. A small CV for IVIs indicates similar time intervals between vowel onsets across the sentence. Here the CV was measured between every other syllable’s vowel onset, corresponding to stressed syllables (see IVI in Figure 2). Once again, a significant difference between conditions was observed, *F*(2,22) = 64.6, *p* < 0.001, *ƞ*^2^ = 0.85. Naturally spoken sentences had the largest timing variations between vowel onsets (M = 0.21), followed by regularly spoken sentences (M = 0.08), while sung sentences showed the smallest variability (M = 0.05). To ensure that the female performer was comparably accurate in timing the sentences with the metronome in the regularly spoken and sung conditions, the relative asynchrony between each vowel onset and the closest metronome pulsation was measured. In this context, a negative mean asynchrony indicates that the vowel onset preceded the metronome tone onset, while a positive asynchrony means that the vowel onset followed the metronome tone (Table 4). There was no significant difference between conditions, indicating similar timing with the metronome in the regularly spoken and sung conditions, *t*(11) = 1.146, *p* = 0.28.

### 2.3. Design and Procedure

Participants performed three tasks. First, they performed a spontaneous tapping task to assess their spontaneous tapping rate (mean and variance) in the absence of a pacing stimulus. They were asked to tap as regularly as possible for 30 seconds, as if they were a metronome or the “tick-tock” of a clock (as in [30]). Participants were asked to tap with the index finger of their dominant hand. Next, participants performed the tapping task with the spoken/sung sentences, as described below. Then the participants repeated the spontaneous tapping task to determine whether their spontaneous rate had changed, and finally, they tapped at a fixed rate with a metronome set to 120 bpm (inter-beat interval of 500 ms) and 96 bpm (inter-beat interval of 625 ms), chosen to match the tempi of the spoken/sung stimuli used in the experiment. The experiment had a total duration of approximately 60 minutes.

In the spoken/sung tapping blocks, each participant was presented with 12 each of naturally spoken sentences, regularly spoken sentences, and sung sentences at the original rate (120 bpm), and six sentences in each condition at the slower rate (96 bpm). These stimuli were mixed and divided into three blocks of 18 trials each. Two pseudo-random orders were created such that not more than two sentences from the same condition occurred consecutively and that the same sentence was never repeated. On each trial, participants first listened to the stimulus; then, for two additional presentations of the same stimulus, they were asked to tap along to the beat that they perceived in the stimulus (as in [56]). The action to perform (listen or tap) was prompted by instructions displayed on a computer screen. Participants pressed a key to start the next trial. Prior to commencing the task, a demonstration video was presented to participants, which showed an individual finger tapping on the sensor with one example stimulus from each condition. In the demonstration, a different sentence was used for each condition, and each was presented at a different rate (84 bpm or 108 bpm) than the ones used in the experiment. The sung sentence example was also presented with a different melody than any heard by participants in the task. After the demonstration, participants completed a practice trial for each type of sentence.

For the metronome task, there were two trials at each metronome tempo (120 bpm and 96 bpm), and the presentation order of the two metronome tempi was counterbalanced across participants. Each metronome stimulus contained sixty 50 ms 440 Hz sine tones. Each metronome trial began with seven tones at the specific tempo, during which participants were instructed to listen and prepare to tap with the metronome. A practice trial was also first performed with a metronome set to 108 bpm. As mentioned previously, since all participants could adapt their tapping rate to the stimuli at both 120 bpm and 96 bpm, only the results of tapping to the metronome at 120 bpm (rate of the original speech stimuli) are reported here.

The experiment took place in a large sound-attenuated studio. The tasks were programed with MAX/MSP (https://cycling74.com). Taps were recorded on a square force-sensitive resistor (3.81 cm, Interlink FSR 406) connected to an Arduino UNO (R3; arduino.cc) running the Tap Arduino script (fsr_silence_cont.ino; [72,73]) and transmitting timing information to a PC (HP ProDesk 600 G1, Windows 7) via the serial USB port. The stimuli were delivered at a comfortable volume through closed headphones (DT 770 PRO, Beyerdynamic, Heilbronn, Germany) controlled by an audio interface (RME Fireface 800). No auditory feedback was provided for participants’ tapping.

### 2.4. Data Analyses

#### 2.4.1. Tapping Data Preprocessing

In the spontaneous tapping task, the first five taps produced were discarded and the following 30 ITIs were used, in line with McAuley et al.’s procedure [30]. If participants produced fewer than 30 taps, the data included all taps produced (the smallest number of taps produced was 16 in this task). Due to recording problems, taps were missing from one beat-deaf participant’s first spontaneous tapping trial.

Recorded taps were first pre-processed to remove ITIs smaller than 100 ms in the spontaneous tapping task, and ITIs smaller than 150 ms in the spoken/sung tapping task and the metronome task. In the three tasks, taps were also considered outliers and were removed if they were more than 50% smaller or larger than the median ITI produced by each participant (median ITI ± (median ITI × 0.5)). Pre-processing of tapping data was based on the procedure described by [74]. Accordingly, the 100 ms criterion was used at first for the spoken/sung task but the number of outliers mean ITIs was high in both groups of participants. A 150 ms criterion was chosen instead considering that it remained smaller than two standard deviations from the average time interval between consecutive syllables across stimuli (M = 245 ms, SD = 47 ms, M − 2SD = 152 ms), thus allowing the removal of more artefact taps while still limiting the risk of removing intended taps. As a result, 1.6% of the taps were removed (range: 0.0–6.4%) in the spontaneous tapping task. In the spoken/sung tapping task, 0.85% of taps per trial were removed (range: 0–36.4% taps/trial). In the metronome task, 5.27% of taps were removed on average (range: 3.4–8.1%), leaving between 54 and 76 taps per trial, of which the first 50 taps produced by each participant were used for analysis.

#### 2.4.2. Analysis of Tapping Data

The mean ITI was calculated for all tapping tasks. In the spoken/sung tapping task, since each participant tapped twice on each utterance in succession, the mean ITIs per stimulus were averaged across the two presentations. However, in 0.16% of the trials, participants did not tap at the same hierarchical level in the two presentations of the stimulus. For example, they tapped on every syllable in the first presentation, and every other syllable in the second presentation. These trials were not included in the calculations of CV, to avoid averaging together taps with differing mean ITIs. Nevertheless, at least 11 of the 12 trials at 120 bpm for each participant in each condition were included in the analyses. In the metronome task, data were also averaged across the two trials with the metronome at 120 bpm.

In the spoken/sung tapping task, inter-tap variability (CV *SD* ITI/mean ITI) was computed for each condition. As Table 4 indicates, the CVs of taps to naturally spoken sentences should be larger than the CVs to regular stimuli. To assess this, we examined how produced ITIs matched the stimulus IVIs (as done by [75,76,77]). ITI deviation was calculated by averaging the absolute difference between each ITI and the corresponding IVI of the stimulus. To control for differences in IVI for each stimulus, the ITI deviation was normalized to the mean IVI of that stimulus and converted to a percentage of deviation (% ITI deviation) with formula (1) below, where x is the current interval and *n* the number of ITI produced:

% ITI deviation = (Ʃ|ITIx – IVIx|/*n*)/mean IVI × 100 (1)

This measure of period deviation gives an indication of how participants’ taps matched the rhythmic structure of the stimuli, whether regular or not.

Period-matching between spoken/sung sentences and taps was further assessed for the stimuli that contained regular beat periods (i.e., regularly spoken, sung, and metronome stimuli) with circular statistics using the Circular Statistics Toolbox for MATLAB [78]. With this technique, taps are transposed as angles on a circle from 0° to 360°, where a full circle corresponds to the period of the IVI of the stimulus. The position of each tap on the circle is used to compute a mean resultant vector. The length of the mean resultant vector (vector length, VL) indicates how clustered the data points are around the circle. Values of VL range from 0 to 1; the larger the value, the more the points on the circle are clustered together, indicating that the time interval between taps matches the IVI of the stimulus more consistently. For statistical analyses, since the data were skewed in the control group for the spoken/sung task (skewness: −0.635, SE: 0.144) and in the metronome tapping task for participants of both groups (skewness: −1.728, SE: 0.427), we used a logit transform of VL (logVL = −1 × log(1 − VL)), as is typically done with synchronization data (e.g., [57,58,60,61,74]). However, for simplicity, untransformed VL is reported when considering group means and individual data. The Rayleigh *z* test of periodicity was employed to assess whether a participant’s taps period-matched the IVI of each stimulus consistently [79]. A significant Rayleigh z test (*p*-value < 0.05) demonstrates successful period matching. An advantage of the Rayleigh test is that it considers the number of taps available in determining if there is a significant direction in the data or not [78]. Using linear statistics, the accuracy of synchronization was further measured using the mean relative asynchrony between taps and beats’ onset time in milliseconds. Note that this measure only included trials for which participants could successfully match the inter-beat interval of the stimuli, as assessed by the Rayleigh test, since the asynchrony would otherwise be meaningless.

The period used to perform the Rayleigh test was adjusted to fit the hierarchical level at which participants tapped on each trial. Since the stimuli had a tempo of 120 bpm (where one beat = two syllables), this meant that if a participant tapped to every word, the period used was 250 ms, if a participant tapped every two words, then 500 ms, and every four words, 1000 ms. This approach was chosen, as suggested by recent studies using circular statistics to assess synchronization to stimuli with multiple metric level (or subdivisions of the beat period), in order to avoid bimodal distributions or to underestimate tapping consistency [61,80,81]. Given this adaptation, in the spoken/sung tapping task, we first looked at the closest hierarchical level at which participants tapped. This was approximated based on the tapping level that fitted best the majority of ITIs within a trial (i.e., the modal tapping level).

#### 2.4.3. Correlation between Pitch Perception and Tapping to Spoken/Sung Sentences

In order to assess the contribution of musical pitch perception to synchronization with the spoken and sung sentences, the scores from the online test of amusia were correlated with measures of tapping variability (CV) and period-matching (% ITI deviation) from the spoken/sung tapping task.

### 2.5. Statistical Analyses

Statistical analyses were performed in SPSS (IBM SPSS Statistics, Armonk, United States, version 24, 2016). A mixed repeated-measures ANOVA with Group as the between-subjects factor was used whenever the two groups were compared on a dependent variable with more than one condition. Because of the small group sample size, a statistical approach based on sensitivity analysis was applied, ensuring that significant effects were reliable when assumptions regarding residuals’ normality distribution and homogeneity of variance were violated [82]. When these assumptions were violated, the approach employed was as follows: (1) inspect residuals to identify outliers (identified using Q—Q plot and box plot), (2) re-run the mixed-design ANOVA without the outliers and assess the consistency of the previous significant results, and (3) confirm the results with a non-parametric test of the significant comparisons [82]. If the effect was robust to this procedure, the original ANOVA was reported. Bonferroni correction was used for post-hoc comparisons. Other group comparisons were performed with Welch’s test, which corrects for unequal variance. Paired *t*-tests were utilized for within-group comparisons on a repeated measure with only two conditions. Effect sizes are reported for all comparisons with *p*-values smaller than 0.50 [83]. To indicate the estimated effect sizes, partial eta-squared values are reported for repeated-measures ANOVA, and Hedge’s g was computed for the other comparisons.

## 3. Results

### 3.1. Spontaneous Tapping

The mean ITI of the spontaneous tapping task ranged from 365 to 1109 ms in control participants and from 348 to 1443 ms in the beat-deaf group (Table 5). There was no significant group difference in the mean ITIs, *F*(1,23) = 1.2, *p* = 0.27, *ƞ^2^* = 0.05, and no significant effect of Time, *F*(1,23) = 0.48, *p* = 0.49, *ƞ^2^* = 0.02, and no interaction, *F*(1,23) = 0.19, *p* = 0.66, indicating that spontaneous tapping was performed similarly before and after the spoken/sung tapping task. In contrast, a main effect of Group emerged in the CV for spontaneous tapping, *F*(1,23) = 18.2, *p* < 0.001, *ƞ^2^* = 0.44, with no effect of Time, *F*(1,23) = 0.30, *p* = 0.59, and no interaction, *F*(1,23) = 0.19, *p* = 0.67. The CV for spontaneous tapping was higher in the beat-deaf group than in the control group (Table 5). As observed by Tranchant and Peretz [63], the beat-deaf individuals showed more inter-tap variability than control participants when trying to tap regularly without a pacing stimulus.

### 3.2. Tapping to Speech and Song

**Figure 3:**
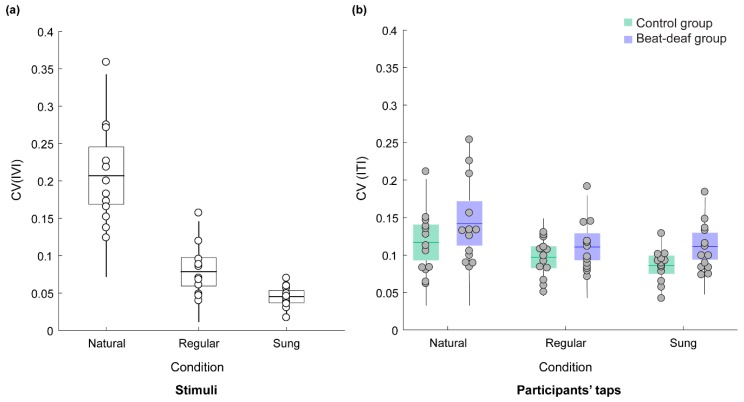
(**a**) Coefficient of variation (CV) of the intervocalic interval (IVI) between stressed syllables of the stimuli. Each dot represents a sentence. (**b**) Mean CV of the inter-tap interval (ITI) produced by the beat-deaf and control group as a function of sentence type. Each dot represents a participant. Boxes correspond to a 95% confidence interval from the mean based on the standard error of the mean (SEM). The darker horizontal line within each box indicates the group mean, while the vertical lines represent two standard deviations from the mean.

As expected, participants’ inter-tap variability (CV) for the naturally spoken sentences was higher than the CV in the other two conditions. Figure 3a depicts the mean CV of the stimulus IVIs and Figure 3b depicts the mean CV for tapping in each condition. The CV for participants’ taps was larger for the naturally spoken sentences (M = 0.13) than for the regularly spoken (M = 0.10) and sung (M = 0.10) sentences, *F*(1.5,35.4) = 15.2, *p* < 0.001, *ƞ^2^* = 0.39. The groups did not differ significantly, *F*(1,24) = 2.8, *p* = 0.10, *ƞ^2^* = 0.11, and there was no interaction with material type, *F*(1.5,35.4) = 0.58, *p* = 0.52. One control participant had a larger tapping CV than the rest of the group for natural speech. Three beat-deaf participants also had larger CVs across conditions. However, removing the outliers did not change the results of the analysis. Thus, the inter-tap variability only discriminated natural speech from the regularly paced stimuli for both groups.

**Figure 4:**
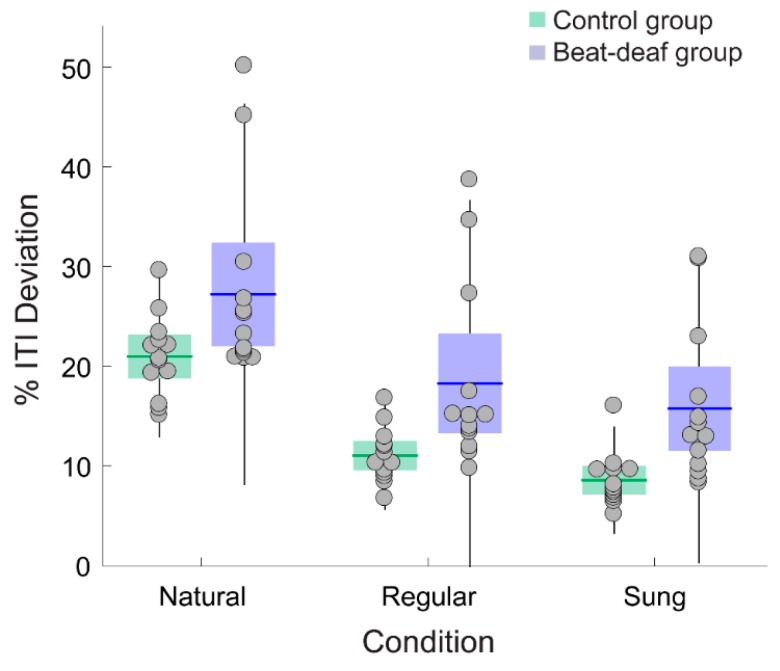
Mean percentage of deviation between the inter-tap intervals (ITIs) produced by each participant and the IVIs of the sentences. Each dot represents a participant. Boxes corresponds to a 95% confidence interval from the mean based on standard error mean (SEM). The black horizontal line within each box indicates the group mean. The vertical lines represent two standard deviations from the mean.

Deviation in period matching between ITIs and IVIs of stimuli indicated that control participants exhibited better performance than beat-deaf participants, whether the stimuli were regular or not. Control participants showed a smaller percentage of deviation between the inter-tap period produced and the corresponding stimulus IVI across stimulus conditions (% ITI deviation; Figure 4), with a main effect of Group, *F*(1,24) = 8.2, *p* = 0.008, *ƞ^2^* = 0.26, a main effect of Material, *F*(1.4,32.5) = 95.9, *p* < 0.001, *ƞ^2^* = 0.80, and no interaction, *F*(1.4,32.5) = 0.19, *p* = 0.74. Post-hoc comparisons showed a significant difference between all conditions: the % ITI deviation was the largest for naturally spoken sentences (20.9% and 27.2% for the control and beat-deaf group, respectively), followed by regular speech (11% and 18.2%) and sung sentences (8.5%, and 15.7%; see Figure 4). These results held even when outliers were removed.

**Figure 5:**
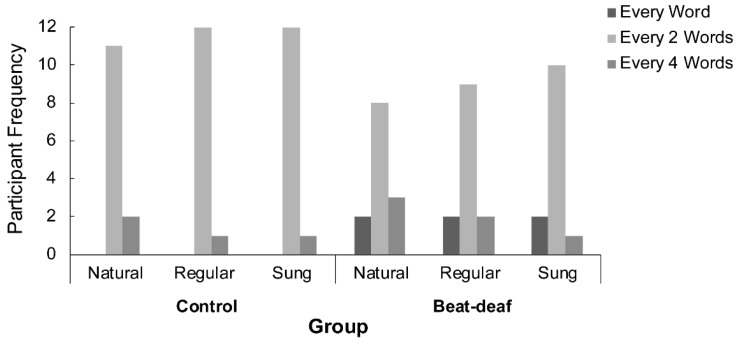
Number of participants in each group who tapped at every word, every two words, or every four words, according to each sentence condition (natural, regular, or sung).

In order to measure synchronization more precisely, we first examined the hierarchical level at which participants tapped. A chi-squared analysis of the number of participants who tapped at each hierarchical level (1, 2, or 4 words) by Condition and Group indicated a main effect of Group, χ²(2,78) = 7.4, *p* = 0.024. In both groups, participants tapped preferentially every two words (see Figure 5), although control participants were more systematic in this choice than beat-deaf participants. Both groups were consistent in the hierarchical level chosen for tapping across conditions. The hierarchical level at which a participant tapped determined the period used in the following analysis of synchronization to the regular stimuli.

The average percentage of trials with successful period matching (using Rayleigh’s z test) for the control group was 91.7% (range: 58–100%) for regularly spoken sentences and 90.4% (range: 50–100%) for sung ones. In the beat-deaf group, the mean percentage of successful period-matched trials was much lower, with 30.4% (range: 0–75%) and 23.8% (range: 0–66.7%) for regularly spoken and sung sentences, respectively. The percentage of trials with successful period matching did not differ between the regular and sung conditions, *t*(25) = 1.297, *p* = 0.21, *g* = 0.10.

We next examined if synchronization was more consistent and accurate for sung than for regularly spoken sentences. These analyses were conducted on trials for which participants were able to synchronize successfully with the beat (i.e., Rayleigh *p*-value < 0.05). Because most beat-deaf participants failed to synchronize with the stimuli, the analyses are limited to the control group. The analyses of the log transform of the mean vector length (logVL) revealed that the control group’s tapping was as constant with regularly spoken sentences (*M* = 1.79, range: 1.18 to 3.09) as with sung ones (*M* = 1.85, range: 1.10 to 3.45), *t*(12) = −0.755, *p* = 0.46, *g* = 0.09. Accuracy of synchronization was assessed with the mean relative asynchrony between taps and beats in milliseconds. Control participants anticipated the beat onsets of sung sentences significantly earlier (M = −14 ms, range: −51 to 19 ms) than the beat onsets of regularly spoken sentences (M = 1 ms, range: −30 to 34 ms), *t*(12) = 3.802, *p* = 0.003, *g* = 0.74. This result suggests that beat onsets were better anticipated in sung sentences than in regularly spoken ones, corroborating results found by Lidji and collaborators [56]. Of note, the two beat-deaf participants (B2 and B4) who could successfully period-match the stimuli on more than 50% percent of trials showed similar consistency (logVL range: 1.08 to 1.53) and accuracy (mean asynchrony range: −3 ms to 22 ms) of synchronization to control participants.

### 3.3. Tapping to Metronome

All participants could successfully match their taps to the period of the metronome, as assessed by the Rayleigh z test, except for one beat-deaf participant (B10) who tapped too fast compared to the 120 bpm tempo (mean ITI = 409 ms for a metronome inter-onset interval of 500 ms). Thus, this participant and a matched control were removed from subsequent analyses in this task. As in previous analyses, control participants had smaller inter-tap variability than beat-deaf participants. This was confirmed by a group comparison with Welch’s test on the CV, *t*(14.0) = 11.698, *p* = 0.004, *g* = 1.35 (control: M = 0.06, SE = 0.003; beat-deaf: M = 0.09, SE = 0.01). Period-matching consistency, using the logVL, also showed a significant group difference, *t*(22.0) = 9.314, *p* = 0.006, *g* = 1.20. The difference between groups was not significant, however, for the mean relative asynchrony between taps and metronome tones, *t*(20.4) = 0.066, *p* = 0.80 (control: M = −56 ms, range: −120 ms to 0 ms; beat-deaf: M = −53 ms, range: −104 ms to −11 ms).

### 3.4. Contribution of Musical Pitch Perception to Entrainment to Utterances

To assess the impact of musical pitch perception on tapping performance, we correlated the scores from the online test of amusia with tapping variability (CV) and period matching (%ITI deviation) for all conditions and participant groups (Table 6). The correlations between CV and musical pitch-related tests did not reach significance, while the % of ITI deviation did for two of the three stimulus conditions when considering participants from both groups. The significant correlation between the Scale test and the % ITI deviation was driven mostly by the beat-deaf group (*r_(8)_* = −0.61) rather than the control group (*r_(11)_* = −0.05). There was also a significant correlation between the Off-key test and % ITI deviation. None of the correlations reached significance with the Off-beat test. However, tapping variability (CV) to sentences and to music (M-BAT) were highly correlated in control but not beat-deaf participants.

These results raise the possibility that beat-deaf individuals with an additional deficit in pitch perception have a more severe impairment in finding the beat. If we compare beat-deaf participants with and without a co-occurring musical pitch deficit, the difference between groups does not reach significance on period-matching consistency of tapping (mean logVL) in the M-BAT beat production test, *t*(6.2) = 1.874, *p* = 0.11, *g* = 1.0. Thus, musical pitch perception seems to have little impact on synchronization to both musical (see [65]) and verbal stimuli.

## 4. Discussion

This study investigated the specialization of beat-based entrainment to music and to speech. We show that a deficit with beat finding initially uncovered with music can similarly affect entrainment to speech. The beat-deaf group, in the current study identified on the basis of abnormal tapping to various pre-existing songs, also show more variable tapping to sentences, whether naturally spoken or spoken to a (silent) metronome, as compared to matched control participants. These results could argue for the domain generality of beat-based entrainment mechanisms to both music and speech. However, even tapping to a metronome or tapping at their own pace is more irregular in beat-deaf individuals than in typical non-musicians. Thus, the results point to the presence of a basic deficiency in timekeeping mechanisms that are relevant to entrainment to both music and speech and might not be specific to either domain.

Such a general deficiency in timekeeping mechanisms does not appear related to an anomalous speed of tapping. The spontaneous tapping tempo of the beat-deaf participants is not different from the tempo of neurotypical controls. What differs is the regularity of their tapping. This anomalous variability in spontaneous tapping has not been reported previously [57,58,59,60,62]. Two beat-deaf cases previously reported from the same lab [62], not included in the present sample, had higher inter-tap variability in unpaced tapping; however, the difference was not statistically significant in comparison to a control group. However, our study includes one of the largest samples of individuals with a beat-finding disorder so far (compared to 10 poor synchronizers in [60] for example), which might explain some discrepancies with previous studies. Only recently (in our lab, [63]) has an anomalously high variability in spontaneous regular tapping in beat-deaf individuals been observed irrespective of the tapping tempo.

A similar lack of precision was noted among beat-deaf participants compared to matched controls when tapping to a metronome, which is in line with Palmer et al. [62]. These similar findings suggest that temporal coordination (both in the presence of auditory feedback from a metronome and in its absence during spontaneous tapping) is impaired in beat-deaf individuals. These individuals also display more difficulty with adapting their tapping to temporally changing signals, such as phase and period perturbations in a metronome sequence [62]. Sowiński and Dalla Bella [60] also reported that poor beat synchronizers had more difficulty with correcting their synchronization errors when tapping to a metronome beat, as reflected in lag -1 analyses. Therefore, a deficient error correction mechanism in beat-deaf individuals may explain the generalized deficit for tapping with and without an external rhythm. This error correction mechanism may in turn result from a lack of precision in internal timekeeping mechanism, sometimes called “intrinsic rhythmicity” [63].

However, the deficit in intrinsic rhythmicity in beat-deaf individuals is subtle. The beat-deaf participants appear sensitive to the acoustic regularity of both music and speech, albeit not as precisely as the control participants. All participants tapped more consistently to regularly spoken and sung sentences than to naturally spoken ones. All showed reduced tapping variability for the regular stimuli, with little difference between regularly spoken and sung sentences, while normal control participants also showed greater anticipation of beat onsets in the sung condition. The latter result suggests that entrainment was easier for music than speech, even when speech is artificially made regular. However, the results may simply reflect acoustic regularity, which was higher in the sung versions than in the spoken versions: the sung sentences had lower intervocalic variability and V-nPVI than regular speech, which may facilitate the prediction of beat occurrences, and, therefore, entrainment. These results corroborate previous studies proposing that acoustic regularity is the main factor supporting entrainment across domains [56].

Another factor that may account for better anticipation of beats in the sung condition is the presence of pitch variations. There is evidence that pitch can influence meter perception and entrainment in music [84,85,86,87,88,89,90,91,92,93]. The possible contribution of musical pitch in tapping to sung sentences is supported by the correlations between perception of musical pitch and period-matching performance (as measured by ITI deviation) in tapping to the sung sentences. However, the correlation was similar for the regularly spoken sentences where pitch contributes little to the acoustic structure. Moreover, the beat-deaf participants who also had a musical-pitch deficit, corresponding to about half the group, did not perform significantly poorer than those who displayed normal musical pitch processing. Altogether, the results suggest that pitch-related aspects of musical structure are not significant factors in entrainment [55,56,94,95].

Thus, a key question remains: What is the faulty mechanism that best explains the deficit exhibited by beat-deaf individuals? One useful model to conceptualize the imprecision in regular tapping that seems to characterize beat deafness, while maintaining sensitivity to external rhythm, is to posit broader tuning of self-sustained neural oscillations in the beat-impaired brain. An idea that is currently gaining increasing strength is that auditory-motor synchronization capitalizes on the tempi of the naturally occurring oscillatory brain dynamics, such that moments of heightened excitability (corresponding to particular oscillatory phases) become aligned to the timing of relevant external events (for a recent review, see [96]). In the beat-impaired brain, the alignment of the internal neural oscillations to the external auditory beats would take place, as shown by their sensitivity to acoustic regularities, but it would not be sufficiently well calibrated to allow precise entrainment.

This account of beat deafness accords well with what is known about oscillatory brain responses to speech and music rhythms [12,97,98,99,100,101,102,103,104]. These oscillatory responses match the period of relevant linguistic units, such as phoneme onsets, syllable onsets, and prosodic cues, in the beta/gamma, theta, and delta rhythms, respectively [16,38,105,106]. Oscillatory responses can also entrain to musical beat, and this oscillatory response may be modulated by the perceived beat structure [81,107,108,109,110,111]. Oscillatory responses may even occur in the absence of an acoustic event on every beat, and not just in response to the frequencies present in the signal envelope, indicating the contribution of oscillatory responses to beat perception [23,25,110]. Ding and Simon [98] propose that a common entrainment mechanism for speech and music could occur in the delta band (1–4 Hz). If so, we predict that oscillations in the delta band would not be as sharply aligned with the acoustic regularities present in both music and speech in the beat-deaf brain as in a normal brain. This prediction is currently under study in our laboratory.

One major implication of the present study is that the rhythmic disorder identified with music extends to speech. This is the first time that such an association across domains is reported. In contrast, there are frequent reports of reverse associations between speech disorders and impaired musical rhythm [112,113,114,115,116]. Speech-related skills, such as phonological awareness and reading, are associated to variability in synchronization with a metronome beat [117,118,119]. Stutterers are also less consistent than control participants in synchronizing taps to a musical beat [120,121]. However, in none of these prior studies [112,116,118] was a deficit noted in spontaneous tapping, hence in intrinsic rhythmicity. Thus, it remains to be seen if their deficit with speech rhythm is related to a poor calibration of intrinsic rhythmicity as indicated here.

The design used in this study, which presented stimuli in the native language of the participants, creates some limitations in generalization of these findings across languages. It is possible, for example, that stress- and syllable-timed languages might elicit different patterns of entrainment [14]. French is usually considered a less “rhythmic” language than English [70,71]. One’s native language has also been shown to influence perception of speech rhythm [14,56,122,123]. For example, Lidji et al. [14] found that tapping was more variable to French sentences than English sentences, and that English speakers tapped more regularly to sentences of both languages. However, using the same protocol as the one used here, Lidji et al. [56] found that tapping was more variable to English than French stimuli, irrespective of participants’ native language. Thus, it is presently unclear whether participants’ native language influence tapping to speech. This should be explored in future studies.

The use of a tapping task has also limited ecological value for entrainment to speech. A shadowing task, for example, where the natural tendency of speakers to entrain to another speaker’s speech rate is measured, could be an interesting paradigm to investigate further entrainment to speech [18,47,48,49] in beat-deaf individuals. The use of behavioral paradigms (tapping tasks), in the absence of neural measurements (such as electroencephalography), leaves open the question of the order in which timing mechanisms contribute to entrainment in speech and music. For example, it is possible that entrainment with music, which typically establishes a highly regular rhythm, is processed at a faster (earlier) timescale than language, which requires syntactic and semantic processing, known to elicit different timescales in language comprehension tasks [124,125]. These questions offer interesting avenues for future directions in comparison of rhythmic entrainment across speech and music.

## 5. Conclusions

In summary, our results indicate that beat deafness is not specific to music, but extends to any auditory rhythm, whether a metronome, speech or song. Furthermore, as proposed in previous studies [55,56], regularity or isochrony of the stimulus period seems to be the core feature through which entrainment is possible.

## Figures and Tables

**Table 1:** Characteristics of the beat-deaf and control groups.

Variables	Beat-deaf (SD) *n* = 13		Control (SD) *n* = 13	
Age (years)	37.4	(17.6)	38.7	(17.8)
Education (years)	18.2	(2.2)	17.6	(3.2)
Musical Training (years)	1.0	(2.3)	1.1	(2.1)
Dance Training (years)	1.3	(3.0)	1.8	(3.1)
WAIS-III Digit Span (ss)	10.0	(3.0)	11.0	(3.0)
WAIS-III Matrix Reasoning (ss) ^a^	13.0	(3.0)	14.0	(1.0)

ss—standard score. ^a^, Scores from 12 beat-deaf and 10 control participants. Some participants did not complete the Matrix Reasoning test because they were Ph.D. students in a clinical neuropsychology program and were too familiar with the test.

**Table 2:** Individual scores of the beat-deaf participants and the group average of their matched controls in the online test of amusia.

	Group															
	Beat-Deaf														Control (*n* = 13)	
Participant	B1	B2	B3	B4	B5	B6	B7 ^†^	B8 ^†^	B9 ^†^	B10 ^†^	B11 ^†^	B12 ^†^	B13 ^†^	M	M	SD
Online test																
Scale (22/30)	23	24	23	23	23	24	21 ^†^	21 ^†^	20 ^†^	22 ^†^	19 ^†^	18 ^†^	22 ^†^	21.8	27.7	2.2
Off-key (16/24)	20	14 ^†^	19	14 ^†^	16 ^†^	14 ^†^	13 ^†^	16 ^†^	15 ^†^	9 ^†^	13 ^†^	14 ^†^	13 ^†^	14.6	19.8	2.2
Off-beat (17/24)	23	21	19	17 ^†^	20	16 ^†^	15 ^†^	17 ^†^	18	17 ^†^	18	18	19	18.3	19.8	1.4

Scores in parentheses represent the cut-off scores taken from Peretz and Vuvan [67]. Participants with co-occurring pitch deafness are marked with ^†^.

**Table 3:** Mean inter-tap interval (ITI) in ms for each group according to material type and tempo.

	Original Tempo		Slowed Tempo	
Condition	Control	Beat-Deaf	Control	Beat-Deaf
Naturally Spoken Sentences (SE)	480.00 (7.70)	489.80 (12.80)	591.77 (8.90)	585.58 (11.70)
Regularly Spoken Sentences (SE)	497.43 (6.52)	522.15 (12.83)	615.20 (4.97)	632.00 (17.69)
Sung Sentences (SE)	488.70 (4.28)	517.08 (13.39)	611.24 (4.12)	664.25 (17.30)

For the comparison of mean ITI between groups and conditions, the mean ITIs were scaled to the ITI corresponding to tapping once every two words (or stressed syllables).

**Table 4:** Stimuli characteristics related to rhythm.

Variable	Naturally Spoken Sentences (SE)		Regularly Spoken Sentences (SE)		Sung Sentences (SE)	
Mean IVI (ms)	458.00	(10.00) *	503.00	( 3.00 )	501.00	(1.000)
V-nPVI	49.40	( 2.50 ) *	42.30	( 2.00 ) *	31.10	(1.800)
CV(IVI)	0.21	( 0.02 ) *	0.08	( 0.01 ) *	0.05	(0.004) *
Beat Asynchrony from Vowel Onset (ms)	-		14.00	(11.00)	−2.00	(5.000)

Values indicate means; standard errors appear in parentheses. IVI—intervocalic interval (in ms); V-nPVI—normalized Pairwise Variability Index for Vocalic Intervals; CV—coefficient of variation (SD IVI/Mean IVI between stressed syllables); Beat asynchrony corresponds to the average of signed values from subtracting metronome tone onset from the closest spoken/sung vowel onset, in milliseconds. *, indicate significant differences.

**Table 5:** Mean inter-tap interval (ITI) and coefficient of variation (CV) of spontaneous tapping.

Group	Spontaneous Tapping-Pre		Spontaneous Tapping-Post	
	Mean ITI ^a^	CV	Mean ITI ^a^	CV
Control (SE)	603 (56)	0.06 (0.003)	565 (57)	0.06 (0.004)
Beat-deaf (SE)	680^b^(47)	0.08^b^ (0.01)	659 (80)	0.08 (0.01)

Numerical values represent group means, with the standard error of the mean in parentheses. ^a^, Values are in milliseconds. ^b^, *n* = 12; otherwise, *n* = 13.

**Table 6:** Spearman correlations between tapping and music perception.

Variable	Scale Test			Off-Key Test			Off-Beat Test			CV M-BAT Production Test		
CV—natural sentences	−0.20 ^a^	−0.10 ^c^	−0.15 ^d^	−0.22 ^a^	−0.06 ^c^	−0.53 ^d^	−0.12 ^a^	0.12 ^c^	−0.25 ^d^	0.47 ^a^	0.78 ^c^	0.14 ^d^
CV—regular sentences	−0.24 ^a^	−0.42 ^c^	−0.27 ^d^	−0.29 ^a^	−0.41 ^c^	−0.48 ^d^	−0.03 ^a^	0.03 ^c^	0.08 ^d^	0.32 ^a^	0.79 ^c^	−0.19 ^d^
CV—sung sentences	−0.26 ^a^	−0.15 ^c^	−0.11 ^d^	−0.35 ^a^	−0.16 ^c^	−0.37 ^d^	−0.24 ^a^	−0.05 ^c^	−0.24 ^d^	0.31 ^a^	0.43 ^c^	−0.11 ^d^
%ITI deviation—natural	−0.30 ^b^	−0.01 ^c^	0.08 ^e^	−0.34 ^b^	−0.39 ^c^	−0.01 ^e^	0.11 ^b^	0.30 ^c^	0.17 ^e^	-	-	-
%ITI deviation—regular	−0.32 ^b^	0.30 ^c^	−0.18 ^e^	−0.55 ^b^	−0.35 ^c^	−0.12 ^e^	−0.19 ^b^	0.05 ^c^	0.17 ^e^	-	-	-
%ITI deviation—sung	−0.54 ^b^	−0.05 ^c^	−0.61 ^e^	−0.43 ^b^	−0.21 ^c^	0.43 ^e^	−0.21 ^b^	0.26 ^c^	0.12 ^e^	-	-	-

CV—coefficient of variation; ITI—inter-tap interval. Outliers from the beat-deaf group were removed, with ^a^ *n* = 24, ^b^ *n* = 23, ^c^ *n* = 13, ^d^ *n* = 11, ^e^ *n* = 10. Columns in white indicate correlations with participants of both groups, light blue with control participants only, and darker blue beat-deaf participants only. Significant correlations after correcting for multiple comparisons are marked in orange (*p* ≤ 0.015).
